# Improved cancer biomarkers identification using network-constrained infinite latent feature selection

**DOI:** 10.1371/journal.pone.0246668

**Published:** 2021-02-11

**Authors:** Lihua Cai, Honglong Wu, Ke Zhou

**Affiliations:** 1 Wuhan National Laboratory for Optoelectronics, School of Computer Science & Technology, Huazhong University of Science & Technology, Wuhan, Hubei, China; 2 School of Mathematics and Computer Science, Guangdong Ocean University, Zhanjiang, Guangdong, China; 3 Shenzhen Genomics Institute, BGI-Shenzhen, Shenzhen, China; Taipei Medical University, TAIWAN

## Abstract

Identifying biomarkers that are associated with different types of cancer is an important goal in the field of bioinformatics. Different researcher groups have analyzed the expression profiles of many genes and found some certain genetic patterns that can promote the improvement of targeted therapies, but the significance of some genes is still ambiguous. More reliable and effective biomarkers identification methods are then needed to detect candidate cancer-related genes. In this paper, we proposed a novel method that combines the infinite latent feature selection (ILFS) method with the functional interaction (FIs) network to rank the biomarkers. We applied the proposed method to the expression data of five cancer types. The experiments indicated that our network-constrained ILFS (NCILFS) provides an improved prediction of the diagnosis of the samples and locates many more known oncogenes than the original ILFS and some other existing methods. We also performed functional enrichment analysis by inspecting the over-represented gene ontology (GO) biological process (BP) terms and applying the gene set enrichment analysis (GSEA) method on selected biomarkers for each feature selection method. The enrichments analysis reports show that our network-constraint ILFS can produce more biologically significant gene sets than other methods. The results suggest that network-constrained ILFS can identify cancer-related genes with a higher discriminative power and biological significance.

## Introduction

The recent development of high-throughput gene expression profiling provided an opportunity for researchers to better understand the molecular characteristics of the cancer disease, leading to advances in its diagnosis and treatment. Accurate identification of the cancer diagnostic biomarkers is very critical for the provision of appropriate therapies and drug development. Some gene mutations, such as BRCA1, BRCA2, VHL, PBMR1 and others were identified to be correlated with an increased tumor aggressiveness in cancer [1–4]. A few targeted therapies have been designed providing more options for treating patients [5]. However, the global incidence and mortality of cancer is still high, 1,806,590 new cancer cases and 606,520 cancer deaths are projected to occur in the United States in 2020 [6]. It is then hoped that ongoing and planned research will develop more reliable and effective feature selection methods to identify more predictors of the tumors’ sensitivity to therapy.

In the field of genomics, disease signatures identification has long been a research topic in which they might revolutionize the way diseases are treated clinically. However, identifying disease associated genes in gene expression data is challenging due to the high dimensional features with low sample size. A lot of studies were published that handled this issue and were employed in the biological analysis [7–12]. From a statistical perspective, it is hard to filter the true factors in high dimensional data [13]. Published material showed that selected features are susceptible to the perturbation of the high dimensional training data. One limitation of these popular methods is that they are merely designed based on statistical or arithmetic points; they don’t utilize any biological information. Over the past few years, more biological knowledge and pathway information became available on the Internet, especially that related to cancer. Some of the biological pathways information can be downloaded from online databases, such as the Kyoto Encyclopedia of Genes and Genomes (KEGG) [14], Reactome [15, 16] and others. These pathways are often presented as graphs where the vertices represent genes or gene products and the edges indicate some regulatory relationship between the genes. Such prior biological information is a very useful supplement to those graph-based feature selection methods. Some popular graph-based methods usually combine *l*_1 penalty with graph regularization procedure to simultaneously obtain sparsity and smoothness for the linear model analysis [9, 17], while other typical methods are designed based on neural networks and deep learning frameworks [18]. However, linear correlation does not often appear in genomic data, thus graph regularization-based models for linear analyses are barely suitable for this task. On the other hand, deep learning frameworks and neural network methods have the limitation that a great number of samples is needed in order to obtain a reliable model while small sample size is a general feature in the field of genomics. In recent years, some achievements have been made on biomarkers identification by integrating biological network into graph algorithms [19–22]. Such methods can produce more robust gene sets across datasets from different cancer types. But it was found that they may find too many hub genes. Furthermore, the significance of genes in such network-based methods is usually evaluated by their neighbors or genes in the same sub-network. Which means that only a limited number of gene subsets with limited cardinality would be tested.

In this article, we propose a novel method by introducing a graph filter procedure on ILFS. ILFS is a graph model-based filter method that was proposed by Giorgio [23]. The motivation behind applying the ILFS method in our work lies in its logic in ranking features, in which the significance of a feature is evaluated by considering all possible subsets of features of any cardinality. Integrating the FIs network with ILFS can utilize the interaction information between genes. In our study, we applied this method to analyze gene expression data for five cancer types: breast invasive carcinoma (BRCA), colon adenocarcinoma (COAD), kidney renal clear cell carcinoma (KIRC), liver hepatocellular carcinoma (LIHC), and prostate adenocarcinoma (PRAD). The proposed method showed improved prediction performance and a much higher selection ratio for known oncogenes than some popular existing methods, including LASSO [7], mRMR [24], VIP score using PLS-DA [25], ReliefF [26] and the original ILFS. We performed functional enrichment analysis on selected markers and found that the number of over-represented GO BP terms obtained from the network-constrained ILFS is much higher than those obtained from other methods. We also performed GSEA on selected biomarkers, the analysis showed that the network-constrained ILFS generates a more biologically significant gene set that is related to the cancer disease than other methods.

## Materials and methods

### Data preprocessing

The data for our research is from The Cancer Genome Atlas (TCGA) platform. The data category we chose is "transcriptome profiling" and the data type is "Gene Expression Quantification". The RNA-Seq expression data of different cancer types is publicly available from (TCGA, https://portal.gdc.cancer.gov/). First, we downloaded the HT-SEQ FPKM (Fragments per Kilobase of transcript per Million mapped reads) values of the type primary solid tumor and solid tissue normal of the BRCA, COAD, KIRC, LIHC, and PRAD cancer types. According to TCGA documentation, the FPKM calculation (1) normalizes read count by dividing it by the gene length and the total number of reads mapped to protein-coding genes. Then we converted the FPKM values to TPM (Transcripts Per Kilobase Million) values as it was shown that TPM values may be more comparable across samples [27]. The conversion follows formula (2). For each sample in our data, 19,754 genes are measured for later analysis.

$$FPKM_{i}=\frac{N_{i}}{M*L_{i}}*10^{9}$$ (1)

*N*_*i*_: Number of reads mapped to the gene *i*

*M*: Number of reads mapped to all protein-coding genes

*L*_*i*_: Length of the gene in base pairs; calculated as the sum of all exons in a gene *i*

**Note**: The read count is multiplied by a scalar (10^9^) during normalization to account for the kilobase and ’million mapped reads’ units.

$$TPM_{i}=\frac{FPKM_{i}}{\sum_{j}FPKM_{j}}\cdot 10^{6}$$ (2)

The datasets include paired and non-paired samples and we divided each dataset into two parts: part one and part two. Part one includes approximately 70% of the paired samples which are used for feature selection. Part two consists of the remaining paired samples (nearly 30% of the paired samples) and all of the non-paired samples, which are used for classifier training and model validation by using a k-fold cross validation process. The "paired" samples mean that the case and control are from different tissues of the same patient. For such patients, the gene expression data of primary solid tumor and normal tissue are available. For other patients, only gene expression data of primary solid tumor are provided, we call them "non-paired" samples. All of "non-paired" samples belong to one group (Tumor). To avoid the effects of genetic differences, we do feature selection only on "paired" samples. More detailed information of the samples and parts is listed in Table 1.

**Table 1:** The number of samples for feature selection and model estimation.

Cancer Type	Part one (Feature Selection)			Part two (Validation)		
	Tumor	Normal	Total	Tumor	Normal	Total
BRCA	80	80	160	1011	33	1044
COAD	28	28	56	428	13	441
KIRC	50	50	100	481	22	503
LIHC	35	35	70	336	15	351
PRAD	36	36	72	458	16	474

Breast invasive carcinoma (BRCA), colon adenocarcinoma (COAD), kidney renal clear cell carcinoma (KIRC), liver hepatocellular carcinoma (LIHC), prostate adenocarcinoma (PRAD).

### Method

We implemented our approach through the following steps: (1) select a subset of differential genes by applying a paired t-test process on the expression data in Part one, (2) select another set of candidates gene according to the number of their connections in the FIs network, (3) combine the above-mentioned two sets and reconstruct the FIs network with candidate genes in the collection, (4) integrate the reconstructed FIs network with ILFS to score the genes. The flow diagram of our method is plotted in S1 Fig.

### Reconstructing the FIs network

Since having a few genes with a very low expression level is statistically meaningless, and performing computations on the whole gene level may be unnecessary, we performed a paired t-test process on the expression data of Part one samples. The cut-off values in the paired t-test process were set as FDR<0.05 and |log_2_ *FC*|>1, so that the top *N* genes that showed great discriminative power were filtered for further feature selection.

We also downloaded the FIs network from the Reactome database, which includes the known pathways in human biology. These pathways are expressed as pairs of genes (*a*_*i*_, *b*_*i*_) and the regulatory relation between them which can be regarded as directed edges in a graph. Since our method is graph-based, we also chose the *M* genes with more than 100 edge connections in the FIs network. We have tested several edge thresholds and found that 100 is applicable in our study. In the genetic network, the more edges a gene has, the more central role it has within the network. We united the sets of *N* genes and *M* genes into a set *T* such that *T* = {*N genes*}∪{*M genes*}. We reconstructed the FIs network *G*_*F*_ as follows: for each edge (*a*_*i*_, *b*_*i*_) in the FIs network, if *a*_*i*_∈*T and b*_*i*_∈*T*, then the edge and its direction are included in *G*_*F*_. We expressed this directed graph *G*_*F*_ as an adjacent matrix *A*_*F*_, according to the direction of the edge (*a*_*i*_, *b*_*i*_) that could be forward, backward or bidirectional, the values of the matrix *A*_*F*_ were assigned using formula (3),

$$\left\{ \begin{array}{l} A_{ij}=1\;if\;gene\;i\;regulate\;gene\;j\\ A_{ij}=0\;otherwise \end{array}\right.$$ (3)

### Network-constrained infinite latent feature selection

ILFS is one kind of filter methods which rank the features depending on the intrinsic properties of the data and are not sensitive to the predictive model type. ILFS ranks the features through three steps: data preprocessing, graph weighting and ranking. In the steps of data preprocessing and ranking, our network-constrained ILFS does the same thing as the original ILFS. The second step of graph weighting includes the introduction of the reconstructed FIs.

Initially, the raw feature space *X* is represented as a set of feature distributions $X=\{\overset{⃑}{x_{1}},\ldots ,\overset{⃑}{x_{n}}\}$, where each *m*×1 vector $\overset{⃑}{x_{i}}$ is the *i*^*th*^ feature (gene) with regard to *m* samples. In the data preprocessing step, a discriminative quantization process is applied on the raw feature distributions $\overset{⃑}{x_{i}}$ through which the raw feature vector $\overset{⃑}{x_{i}}$ is mapped to a countable nominal smaller set of intervals and represented as a descriptor *f*_*i*_, where *f*_*i*_ is a *t*×1 vector (*t*≪*m*); thus, each feature will be represented by a new low-dimensional pattern. Following this formulation, a strong new representation of the training data *X* in the form of *F* = {*f*_1_,…,*f*_*n*_} where each feature *f*_*i*_ is described using a vocabulary of few tokens is obtained.

In the graph-weighting process, an undirected fully connected graph *G* is built whose nodes correspond to each feature *f*_*i*_ and whose edges are weighted by *f*_*i*_⇝*f*_*j*_, which represents the probability that the features *x*_*i*_ and *x*_*j*_ are relevant. Using a learning framework that is based on a variation of the probabilistic latent semantic analysis (PLSA) technique, the weights were computed by modeling the probability of each co-occurrence in *f*_*i*_, *f*_*j*_. After this process, we obtained the weighted graph *G*, which can be expressed as an adjacent matrix *A*_*p*_. We have intuitively found that ILFS constructs a fully connected graph in this step that assumes all features to be connected with each other, while in fact the true correlation structure of the gene expression data is much sparer than this. This enlightened us that incorporating a prior knowledge of genetic pathways may produce more biologically reasonable results, and we proposed to add an extra process after the graph weighting step. We employed the reconstructed graph matrix *A*_*F*_ as a filter to achieve a sparser connection graph. We implemented this process by calculating the following formula:

$$A=A_{p}\circ A_{F}$$ (4)

where the symbol ∘ denotes a Hadamard (element-wise) product. The Hadamard product is a binary operation that takes two matrices of the same dimensions, and produces another matrix where each element *A*_*ij*_ is the product of (*A*_*p*_)_*ij*_ and (*A*_*F*_)_*ij*_. The value of *A*_*ij*_ will be zero after this operation if there is no regulatory relationship between the genes *i* and *j*. As a result, only actual pathways are retained after this process.

In ranking step, the importance score of a feature is defined as a function of the importance of its neighbors. Let *γ* = {*υ*_0_ = *i*, *v*_1_, *v*_2_,…,*v*_*l*−1_, *v*_*l*_ = *j*} denote a path of length *l* between node *i* and *j*, namely, feature ${\overset{⃑}{x}}_{i}$ and ${\overset{⃑}{x}}_{j}$, through other nodes *v*_1_, *v*_2_,…,*v*_*l*−1_. Suppose that the length *l* is lower than the total number of nodes in the graph. Therefore, a path is a subset of available features(nodes). The join probability that *γ* is a good subset of features can be estimated as

$$P_{\gamma }=\prod_{k=0}^{l-1}A_{v_{k},v_{k+1}}$$ (5)

Let a set $P_{i,j}^{l}$ as considering all the paths of length *l* between *i* and *j*. The energy of all the paths of length *l* can be summed as follows,

$$C_{l}(i,j)=\sum_{\gamma \in {\mathbb{P}}_{i,j}^{l}}P_{\gamma }$$ (6)

Following the standard matrix algebra, it can be written as:

$$C_{l}\left(i,j\right)=A^{l}\left(i,j\right)$$ (7)

Considering all the possible subsets of features of any cardinality means considering all the possible paths of any length in the graph. As a result, extending the path length to infinite implies calculating the geometric series of matrix *A*.

$$\hat{C}=\overset{\infty }{\underset{l=1}{\sum }}A^{l}$$ (8)

Tending the path length to infinite brings divergence. So that a consistence *r* for regularization is assigned for the computation.

$$\overset{ˇ}{C}=\overset{\infty }{\underset{l=1}{\sum }}r^{l}A^{l}$$ (9)

From an algebraic point of view, *Č* can be efficiently computed by using the convergence property of the geometric power series of a matrix. Therefore, the value of *Č* can be calculated as follows:

$$\overset{ˇ}{C}={\left(I-\gamma A\right)}^{-1}-I$$ (10)

Matrix *Č* encodes all the information about the goodness of the set of features. The final scores for each node can then be obtained by marginalizing the quantity š(*i*) = [*Če*]_*i*_, where *e* denotes a 1D array of ones. Ranking the š(*i*) scores in a descending order, we can get the most discriminative features at the top of the sorted list.

### Accuracy of predictors

**Fig 1:**
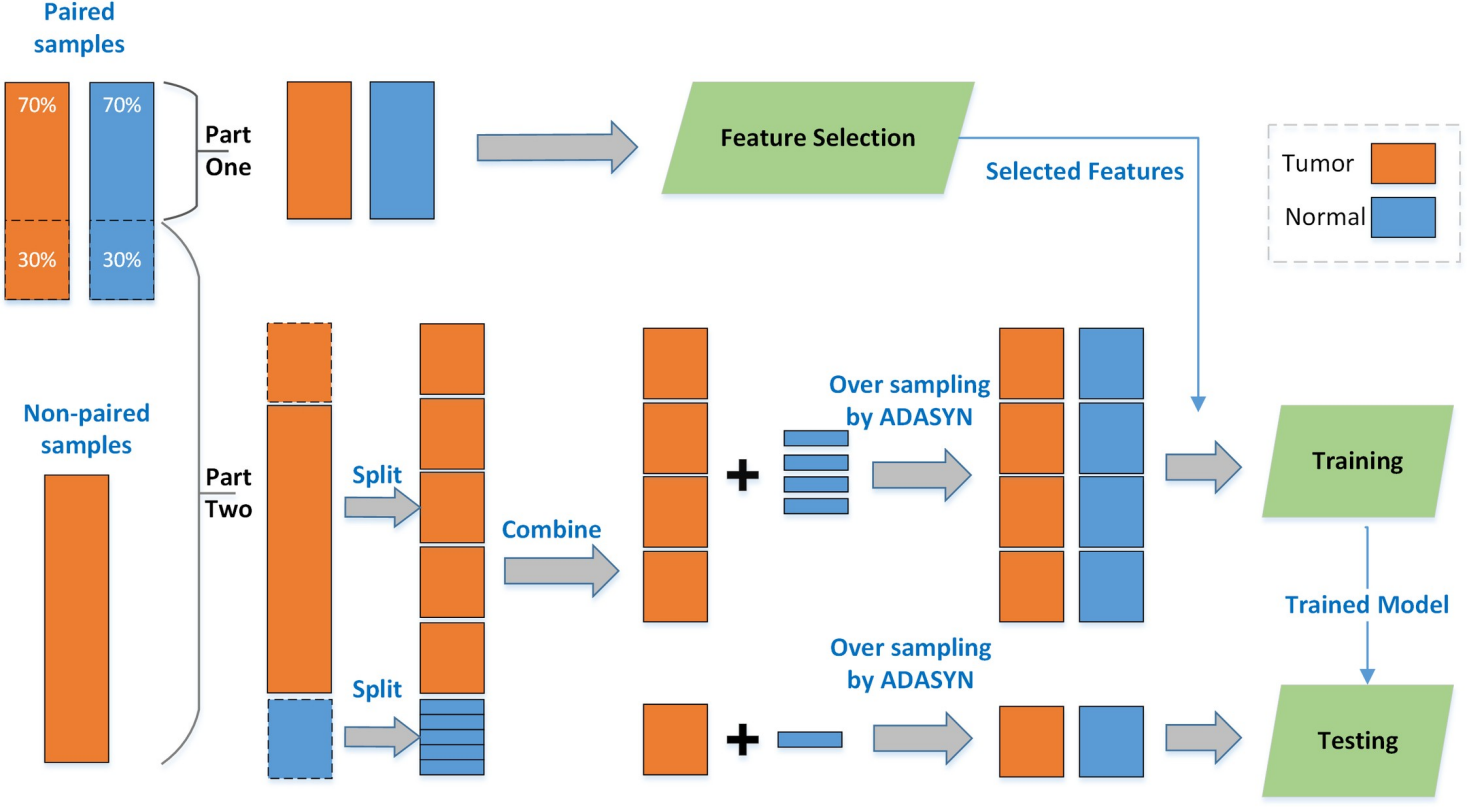
Data division, splitting, combination, training and testing.

In order to measure the prediction performance of our proposed method, the training data reduced to selected genes was used to train the classifiers which were subsequently employed to be tested on the rest of samples. We performed the model training and validation process on the samples in part two. As can be seen in Table 1, the amount of the available normal samples is much less than the tumor samples. As most standard classifiers assume a relatively balanced class distribution, training with imbalanced data will lead to illusive classification performance. Therefore, we adopted a technique called ADASYN sampling approach for imbalanced training [28]. The basic idea of ADASYN is to adaptively generate more synthetic data for the minority class samples according to their distributions. The characteristic of this method is that it can shift the decision boundary to focus on those samples that are hard to be learned. By applying ADASYN, the ratio of normal to tumor samples was adjusted to be close to 1. To avoid over-fitting, the generation of training and testing data was separately executed in the classification and validation process. We tested support vector machine (SVM) classifier on the expression data restricted to the top 100 genes. We implemented a 5-fold CV process on the generative datasets for 100 times. To explore the prediction performance, we employed the mean AUC value over 100 times CV as the measurement for the selected features. To better understand the data processing flow, we plotted it in **Fig 1**.

## Results

### Prediction of selected biomarkers

**Fig 2:**
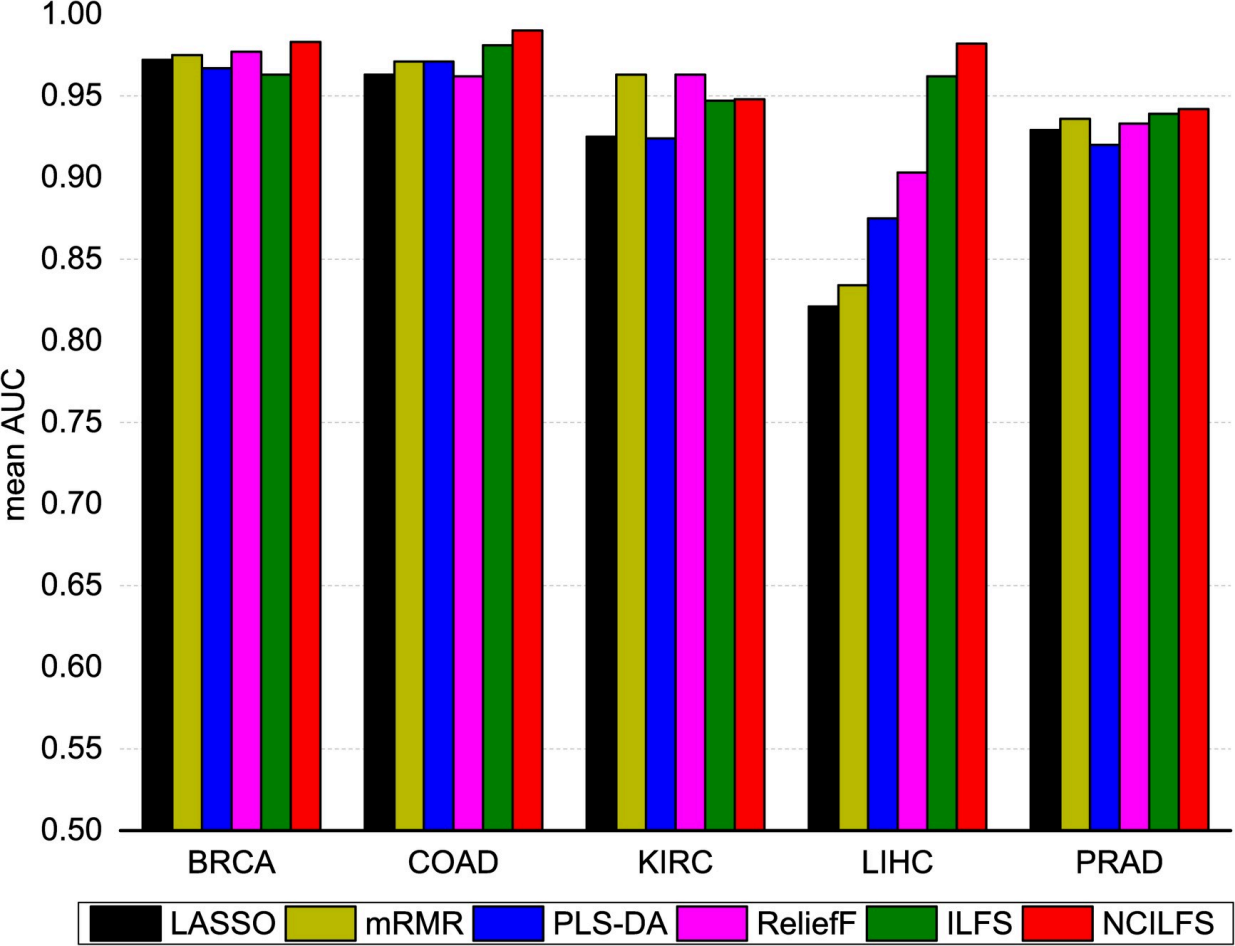
Prediction accuracy in mean AUCs obtained by LASSO, mRMR, ILFS, and Network-Constrained ILFS (NCILFS) for breast invasive carcinoma (BRCA), colon adenocarcinoma (COAD), kidney renal clear cell carcinoma (KIRC), liver hepatocellular carcinoma (LIHC), prostate adenocarcinoma (PRAD).

**Fig 3:**
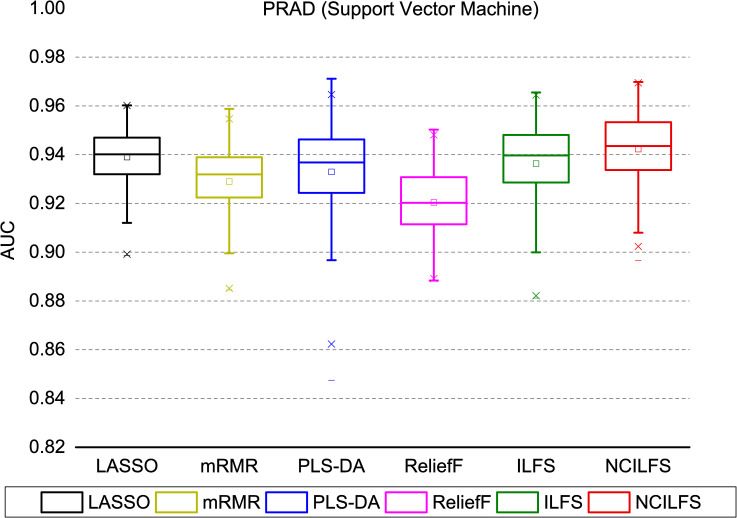
AUC distribution obtained by SVM as a classifier trained on features from LASSO, mRMR, ILFS, Network-Constrained ILFS (NCILFS), VIP score using PLS-DA and ReliefF for PRAD.

To evaluate the prediction performance, several popular feature selection methods, including mRMR, LASSO, PLS-DA and ReliefF and the original ILFS were compared with the proposed method. We provided the top 100 biomarkers obtained by the above-mentioned feature selection methods for five cancer types in the S2 Table. Appropriate selection of the tuning parameter in penalized likelihood methods is very essential for high dimensional data analysis; thus, we executed an additional procedure for LASSO to select the optimal tuning parameter [29]. We tested the accuracy of the top 100 genes obtained by each feature selection method, combined with an SVM classifier to train the prediction models. We repeated a 5-fold cross-validation process 100 times and computed the average AUC value. We used the following parameters for SVM (linear kernel, C = 1); we have also tested a few parameters and found that no significant better results were reached. The mean AUCs over 100 times classifications limited to top 100 genes for five cancer types were plotted in **Fig 2**. Generally, we observed that the network-constrained ILFS shows better prediction performance, except in the case of KIRC. For KIRC, mRMR and ReliefF had the greatest predictive power. Moreover, network-constrained ILFS showed much better performance for LIHC than other cancers. To explore the distribution of AUCs, we also plotted the accuracies obtained by each feature selection method on the PRAD data set as shown in **Fig 3**. Another measurement to assess these feature selection methods is the *F*1 score. The *F1* score is a weighted average of precision and recall, which can be calculated by formula (11). The results are listed in S1 Table.

$$F1=2*\frac{precision*recall}{\left(precision+recall\right)}$$ (11)

### Known oncogenes including test

**Fig 4:**
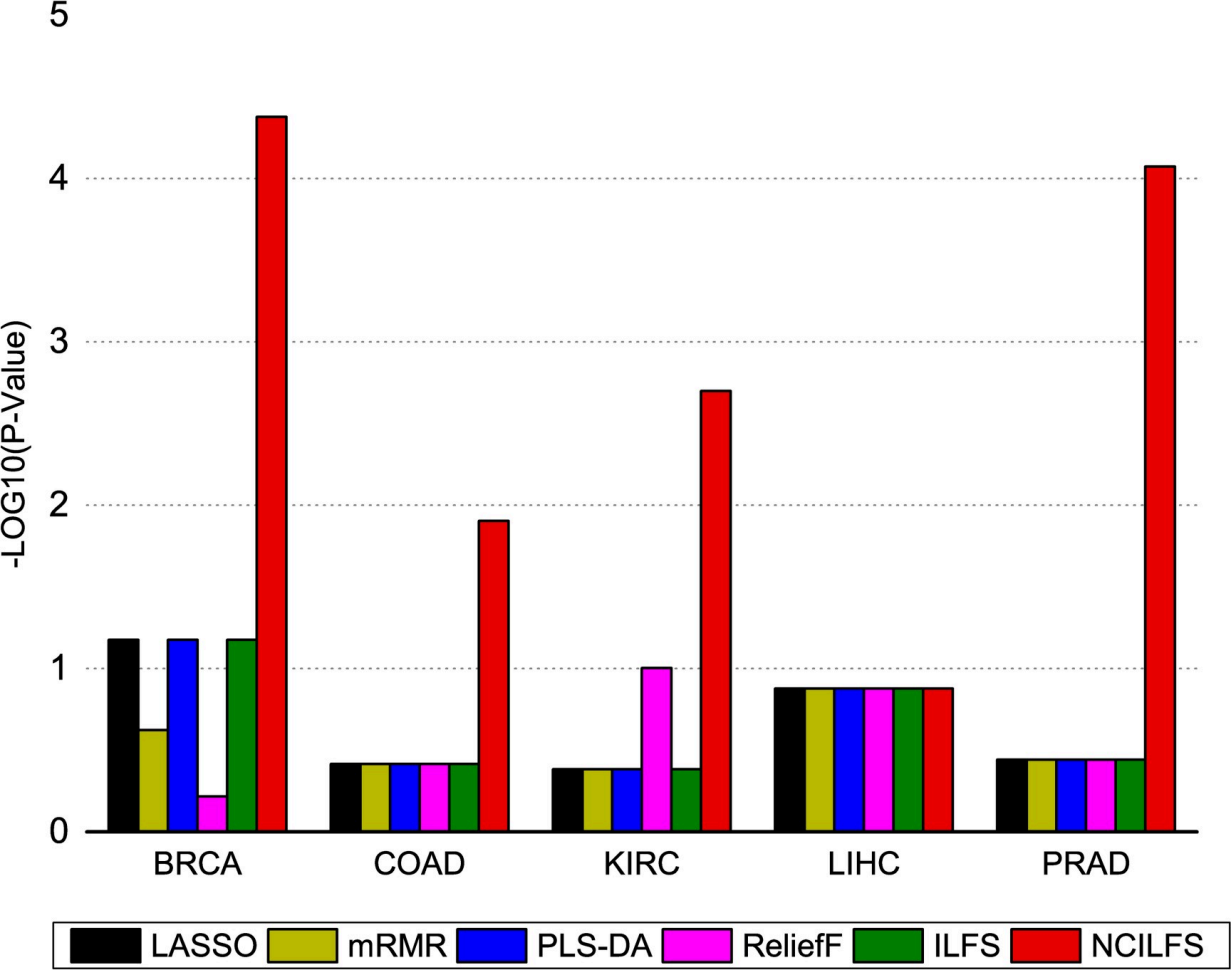
The–log10 (p-value) of selected oncogenes using hyper geometric tests, these genes are identified by LASSO, mRMR, ILFS, Network-constrained ILFS (NCILFS), VIP score using PLS-DA, and ReliefF for BRCA, COAD, KIRC, LIHC, and PRAD.

The efforts of many scientists resulted in revealing some genetic mutations that might be involved in cancer development. The IntOGen-mutations platform summarizes the somatic mutations, genes and pathways that are involved in tumor genesis [30]. We collected the known oncogenes of BRCA, COAD, KIRC, LIHC, and PRAD from this platform and counted the number of known oncogenes in the top 100 genes obtained by these feature selection methods and then calculated the p-values using hyper geometric tests. The number of oncogenes in selected 100-genes is listed in S3 Table. **Fig 4** shows the significance values of the selected oncogenes ratio for each method on five cancer types using hyper geometric tests. It is obvious that the proposed method outperforms other methods in this task. The selected oncogenes by the network-constrained ILFS are listed in Table 2. The results show that our network-constrained ILFS has a great chance for BRCA, COAD, KIRC and PRAD to mine the true factors in high dimensional gene expression data. For LIHC, no oncogenes in top 100 have been detected from any feature selection method.

**Table 2:** The selected known oncogenes for five cancer types by the network-constrained ILFS.

Cancer Type	Selected oncogenes
BRCA	HSPA8 AQR POLR2B CCAR1 FUS DHX15
COAD	PCBP1 POLR2B
KIRC	DHX15 FUS CCAR1
LIHC	
PRAD	FIP1L1 PRPF8 AQR HSPA8

### Biological interpretability

**Fig 5:**
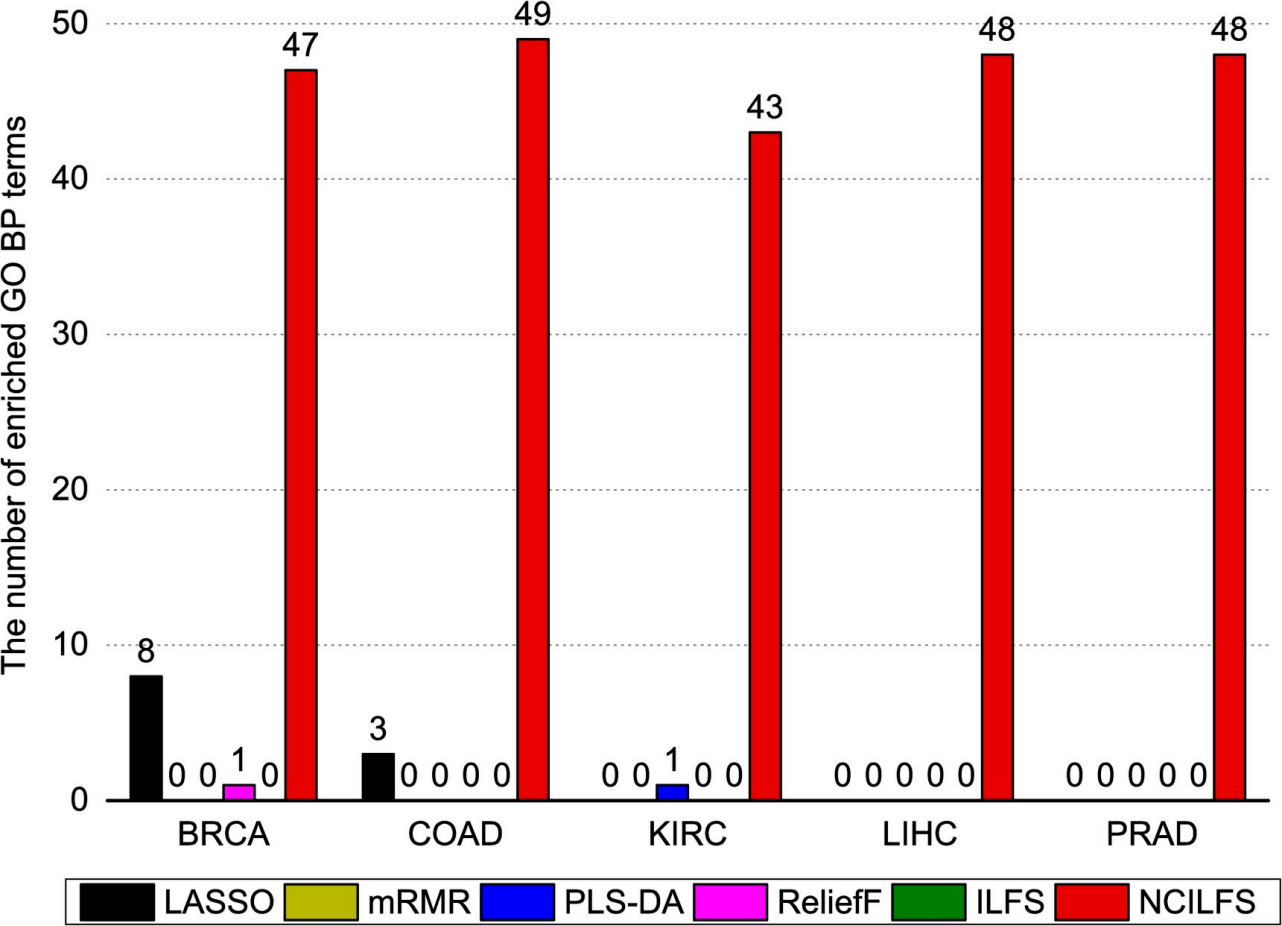
The number of enriched GO BP terms overrepresented at 5% FDR.

Finding gene groups that show predictive power is no longer a very hard job. However, mining biomarkers that provide insights into the biological mechanisms remains a challenge. To assess the interpretability significance of selected biomarkers, we adopted two ways: GO functional enrichment analysis and GSEA [31]. The top 100 biomarkers gene list was analyzed using the tool DAVID [32] to produce GO BP terms. We computed the number of GO BP terms that are overrepresented at 5% FDR. **Fig 5** shows the number of enriched GO BP terms for five cancer types. The detailed information of the GO functional enrichment analysis is listed in the S4–S8 Tables. In general, the number of overrepresented GO terms indicates how easily selected biomarkers can extract a biological insight. Apparently, the network-constrained ILFS provides a more functionally significant gene set. We also applied a gene set enrichment analysis to the top 100 biomarkers obtained from each feature selection method. The reference gene sets that were used in the GSEA process were downloaded from the Molecular Signatures Database [33]. We chose the C4 and C6 collections as the reference gene sets which includes computational gene sets that were defined by mining large collections of cancer-oriented microarray data and oncogenic signature gene sets that were directly from microarray gene expression data from cancer gene perturbations. The normalized enrichment score (NES) is the primary statistic for examining gene set enrichment results, which reflects the degree to which a gene set in the C4 and C6 collections is overrepresented at the top or the bottom of the selected biomarkers ranked list. FDR is the estimated probability of a gene set with a given NES, and the nominal p-value estimates the statistical significance of the enrichment score for a single gene set. In general, an FDR cutoff of 25%, |NES|>1 or a nominal p value cutoff of 5% are appropriate. The GSEA analysis report about PRAD is shown in Table 3. Obviously, we observed that the number of significantly enriched gene sets obtained by the network-constrained ILFS is much larger than the number of those obtained by other methods. Typically, the larger the number of significantly enriched gene sets is, the more likely interesting hypothesis will generate. The results indicate that the network-constrained ILFS could produce more biological interesting gene set than other methods. The detailed GSEA analysis report for five cancer types is provided in S9 Table.

**Table 3:** Summary report about gene set enrichment analysis (GSEA) for PRAD.

Method	Summary Report
NCILFS	**103 / 110** gene sets are upregulated in phenotype Tumor
	**23** gene sets are significant at FDR < 25%
	**2** gene sets are significantly enriched at nominal pvalue < 1%
	**13** gene sets are significantly enriched at nominal pvalue < 5%
ILFS	15 / 29 gene sets are upregulated in phenotype Tumor
	0 gene sets are significant at FDR < 25%
	0 gene sets are significantly enriched at nominal pvalue < 1%
	0 gene sets are significantly enriched at nominal pvalue < 5%
LASSO	11 / 25 gene sets are upregulated in phenotype Tumor
	0 gene sets are significant at FDR < 25%
	0 gene sets are significantly enriched at nominal pvalue < 1%
	0 gene sets are significantly enriched at nominal pvalue < 5%
mRMR	4 / 9 gene sets are upregulated in phenotype Tumor
	0 gene sets are significant at FDR < 25%
	0 gene sets are significantly enriched at nominal pvalue < 1%
	0 gene sets are significantly enriched at nominal pvalue < 5%
PLS-DA	11 / 34 gene sets are upregulated in phenotype Tumor
	0 gene sets are significant at FDR < 25%
	0 gene sets are significantly enriched at nominal pvalue < 1%
	0 gene sets are significantly enriched at nominal pvalue < 5%
ReliefF	19 / 35 gene sets are upregulated in phenotype Tumor
	0 gene sets are significant at FDR < 25%
	0 gene sets are significantly enriched at nominal pvalue < 1%
	0 gene sets are significantly enriched at nominal pvalue < 5%

From GO BP enrichment analysis results, we found that the selected genes by our method for five cancer types are significantly involved in GO:0000398~mRNA splicing, via spliceosome, GO:0010467~gene expression, GO:0008543~fibroblast growth factor receptor signaling pathway, and GO:0006370~7-methylguanosine mRNA capping. The core spliceosome machinery has been demonstrated to be overexpressed in multiple cancers and affect autophagy and cell proliferation, becoming a potential therapeutic target for malignant solid tumors treating [34, 35]. The fibroblast growth factor receptor (FGFR) pathway is increasingly proved to play a role in the pathogenesis of different tumor types, such as urothelial, breast, endometrial, squamous cell lung cancer and hepatocellular carcinoma [36–38]. These facts confirm our method in biological interpretations.

In GSEA analysis, the most significantly enriched gene sets from our method include MORF_SOD1 (Neighborhood of superoxide dismutase 1 in the MORF expression compendium), MORF_CSNK2B (Neighborhood of casein kinase 2, beta polypeptide in the MORF expression compendium) for prostate cancer, GCM_CSNK2B (Neighborhood of casein kinase 2, beta polypeptide in the GCM expression compendium) and MORF_EIF3S2 (Neighborhood of eukaryotic translation initiation factor 3, subunit 2 beta, 36kDa in the MORF expression compendium) for breast cancer. Casein kinase 2 (CK2) is a ubiquitous serine/threonine protein kinase. A previous study has demonstrated that CK2 is to be overexpressed in a number of human cancers, including prostate and breast cancer [39, 40]. SOD1, plays an important role in maintaining the normal life activities of cells, which has been reported associated with tumorigenesis [41, 42]. Eukaryotic initiation factor 3 (EIF3) is involved in the initiation process of protein translation and overexpression of its subunit eukaryotic translation initiation factor 3 (EIF3I) has been observed in breast carcinoma [43]. The results of GSEA also prove that the proposed method can identify genes with biological significance.

## Discussion

In the field of genomics, it is very common to have a high dimensional data with low sample size; thus, feature selection plays a very critical role in scientific discoveries. Most existing feature selection methods rank the features only from the statistical perspective. Such methods tend to filter out those genes that show the best discriminative power in model training. However, a lot of those genes are meaningless when it comes to the biological process and interpretability. This can be perceived from our experiments result. It is easy to find in S3 Table that the proposed method can produce highly overlapping signatures over all cancer types, while classical methods fail to identify common gene sets across the same cancer types. For future work, it is more promising to explore such similar signatures than those no overlapping signatures. LASSO, mRMR, ILFS, VIP score using PLS-DA and ReliefF show no significant worse performance than the network-constrained ILFS regarding the prediction accuracy, but the signatures obtained by them share little overlap not only with each other but also with known oncogenes. This demonstrates that different gene groups can lead to same predictive accuracies, but methods with great power in model training are not necessarily good at selecting true features. It implies that maybe no biological insight should be expected from the analysis of those genes using such methods. To avoid selecting too many differential but biologically meaningless genes, we propose that adding some biological prior information may improve the reliability and feasibility of statistical methods. For this purpose, we employed the FIs network to modify the ILFS graph-weighting process. In addition, we followed a special way in the initial gene screening step. We picked two kinds of genes at first. One kind is differential on expression data obtained by a paired-t test process. Another is central in the graph which is measured by its connections. This setting is very important because the basic idea of ILFS is to consider all possible subsets, which can be regarded as walking down all possible paths in a graph, while the central genes are key nodes to connect those paths. As a result, the selected biomarkers showed both great prediction power and remarkable biological significance.

## Conclusions

In this study, we proposed a novel feature selection method which combined the biological network with the statistical method of ILFS. We applied this method to identify biomarkers in the gene expression data of BRCA, COAD, KIRC, LIHC, and PRAD. First, we compared it with the methods of ILFS, mRMR, LASSO, VIP score using PLS-DA and ReliefF on estimation precision and selection ratio of known oncogenes. Then, we performed functional enrichment and gene set enrichment analysis on selected features and perceived that the selected features are meaningful from a biological perspective. The results indicate that the network-constrained ILFS is helpful in cancer biomarkers identification.

## Supporting information

S1 Fig The flow diagram of network-constrained infinite latent feature selection. (TIF)

S1 Table The F1 score obtained by six feature selection methods. (XLSX)

S2 Table Biomarkers selected for BRCA, COAD, KIRC, LIHC and PRAD. (XLSX)

S3 Table The number of selected oncogenes. (XLSX)

S4 Table GO BP enrichment analysis of biomarkers for BRCA. (XLSX)

S5 Table GO BP enrichment analysis of biomarkers for COAD. (XLSX)

S6 Table GO BP enrichment analysis of biomarkers for KIRC. (XLSX)

S7 Table GO BP enrichment analysis of biomarkers for LIHC. (XLSX)

S8 Table GO BP enrichment analysis of biomarkers for PRAD. (XLSX)

S9 Table Summary report of gene set enrichment analysis (GSEA). (XLSX)

## References

[1] Roa B. B., Boyd A. A., Volcik K., & Richards C. S. (1996). Ashkenazi jewish population frequencies for common mutations in brca1 and brca2. Nature Genetics, 14(2), 185–187. 10.1038/ng1096-185 8841191

[2] Foster K., Prowse A., Van d. B. A., Fleming S., Hulsbeek M. M., & Crossey P. A., et al (1994). Somatic mutations of the von hippel-lindau disease tumour suppressor gene in non-familial clear cell renal carcinoma. Human Molecular Genetics, 3(12), 2169–2173. 10.1093/hmg/3.12.2169 7881415

[3] Zbar B., Brauch H., Talmadge C., & Linehan M. (1987). Loss of alleles of loci on the short arm of chromosome 3 in renal cell carcinoma. Nature, 327(6124), 721–724. 10.1038/327721a0 2885753

[4] Guo G., Gui Y., Gao S., Tang A., Hu X., & Huang Y., et al (2012). Frequent mutations of genes encoding ubiquitin-mediated proteolysis pathway components in clear cell renal cell carcinoma. Nature Genetics, 44(1), 17–9. 10.1038/ng.1014 22138691

[5] Singer E. A., Gupta G. N., & Srinivasan R. (2011). Update on targeted therapies for clear cell renal cell carcinoma. Current Opinion in Oncology, 23(3), 283–9. 10.1097/CCO.0b013e32834479c0 21330923 PMC3488422

[6] Siegel R.L., Miller K.D. and Jemal A. (2020), Cancer statistics, 2020. CA A Cancer J Clin, 70: 7–30. 10.3322/caac.21590 31912902

[7] Tibshirani R. Regression shrinkage and selection via the lasso. Journal of the Royal Statistical Society Series B (Methodological). 1996; p. 267–288.

[8] Zou H., & Hastie T. (2005). Regularization and variable selection via the elastic net. Journal of The Royal Statistical Society Series B-statistical Methodology, 67(2), 301–320.

[9] Li C., & Li H. (2008). Network-constrained regularization and variable selection for analysis of genomic data. Bioinformatics, 24(21), 2566 10.1093/bioinformatics/btn412 18310618

[10] Wan S., Mak M. W., & Kung S. Y. (2015). Mem-mEN: predicting multi-functional types of membrane proteins by interpretable elastic nets. IEEE/ACM transactions on computational biology and bioinformatics, 13(4), 706–718. 10.1109/TCBB.2015.2474407 26336143

[11] Guyon I., Weston J., Barnhill S. et al Gene Selection for Cancer Classification using Support Vector Machines. Machine Learning 46, 389–422 (2002). 10.1023/A:1012487302797

[12] Wan S., Mak M. W., & Kung S. Y. (2016). Sparse regressions for predicting and interpreting subcellular localization of multi-label proteins. BMC bioinformatics, 17(1), 97 10.1186/s12859-016-0940-x 26911432 PMC4765148

[13] Fan J, Lv J. Sure independence screening for ultrahigh dimensional feature space. Journal of the Royal Statistical Society: Series B (Statistical Methodology). 2008; 70(5):849–911. 10.1111/j.1467-9868.2008.00674.x 19603084 PMC2709408

[14] Kanehisa M., Furumichi M., Tanabe M., Sato Y., & Morishima K. (2017). Kegg: new perspectives on genomes, pathways, diseases and drugs. Nucleic Acids Research, 45(D1), D353–D361. 10.1093/nar/gkw1092 27899662 PMC5210567

[15] Croft D., Mundo A. F., Haw R., Milacic M., & D’Eustachio P. (2014). The reactome pathway knowledgebase. Nucleic Acids Research, 42(D1). 10.1093/nar/gkt1102 24243840 PMC3965010

[16] Fabregat A., Jupe S., Matthews L., Sidiropoulos K., Gillespie M., Garapati P., et al (2018). The Reactome Pathway Knowledgebase. Nucleic Acids Research, 46(D1), D649–D655. 10.1093/nar/gkx1132 29145629 PMC5753187

[17] Park M. Y., & Hastie T. (2008). Penalized logistic regression for detecting gene interactions. Biostatistics, 9(1), 30–50. 10.1093/biostatistics/kxm010 17429103

[18] Lin E., Kuo P. H., Liu Y. L., Yu W. Y., Yang A. C., & Tsai S. J. (2018). A deep learning approach for predicting antidepressant response in major depression using clinical and genetic biomarkers. Frontiers in Psychiatry, 9, 290–. 10.3389/fpsyt.2018.00290 30034349 PMC6043864

[19] Kim M., Oh I., & Ahn J. (2018). An improved method for prediction of cancer prognosis by network learning. Genes, 9(10). 10.3390/genes9100478 30279327 PMC6210393

[20] Winter C., Kristiansen G., Kersting S., Roy J., & Robert Grü Tzmann. (2012). Google goes cancer: improving outcome prediction for cancer patients by network-based ranking of marker genes. Plos Computational Biology, 8(5), e1002511 10.1371/journal.pcbi.1002511 22615549 PMC3355064

[21] Roy J., Winter C., Isik Z., & Schroeder M. (2014). Network information improves cancer outcome prediction. Briefings in Bioinformatics (4), 612–25. 10.1093/bib/bbs083 23255167

[22] Barter R. L., Schramm S. J., Mann G. J., & Yang Y. H. (2014). Network-based biomarkers enhance classical approaches to prognostic gene expression signatures. BMC Systems Biology, 8(Suppl 4), 1–16. 10.1186/1752-0509-8-S4-S5 25521200 PMC4290694

[23] Roffo G., Melzi S., Castellani U., & Vinciarelli A. (2017). Infinite latent feature selection: a probabilistic latent graph-based ranking approach. 10.1109/ICCV.2017.156 32750789

[24] Peng H., Long F., & Ding C. (2005). Feature selection based on mutual information: criteria of max-dependency, max-relevance, and min-redundancy. IEEE Transactions on Pattern Analysis and Machine Intelligence, 27(8), 1226–1238. 10.1109/TPAMI.2005.159 16119262

[25] Tran T. N., Afanador N. L., Buydens L. M. C., & Blanchet L. (2014). Interpretation of variable importance in partial least squares with significance multivariate correlation (smc). Chemometrics and Intelligent Laboratory Systems, 138, 153–160.

[26] Kononenko I. a. Š. E. a. R.-S. M. (1997). Overcoming the Myopia of Inductive Learning Algorithms with RELIEFF. Applied Intelligence, 7, 39–55. 10.1023/A:1008280620621

[27] Rahman M., Jackson L. K., Johnson W. E., Li D. Y., Bild A. H., & Piccolo S. R. (2015). Alternative preprocessing of RNA-Sequencing data in The Cancer Genome Atlas leads to improved analysis results. Bioinformatics, 31(22), 3666–3672. 10.1093/bioinformatics/btv377 26209429 PMC4804769

[28] He, H., Bai, Y., Garcia, E. A., & Li, S. (2008). ADASYN: Adaptive Synthetic Sampling Approach for Imbalanced Learning. Neural Networks, 2008. IJCNN 2008. (IEEE World Congress on Computational Intelligence). IEEE International Joint Conference on. IEEE.

[29] Fan Yingying, and Cheng Yong Tang. Tuning parameter selection in high dimensional penalized likelihood. Journal of the Royal Statistical Society: Series B (Statistical Methodology) 75.3 (2013): 531–552.

[30] Gonzalezperez A., Perezllamas C., Deupons J., Tamborero D., Schroeder M. P., & Jenesanz A., et al (2013). Intogen-mutations identifies cancer drivers across tumor types. Nature Methods, 10(11), 1081–1082. 10.1038/nmeth.2642 24037244 PMC5758042

[31] Subramanian A., Tamayo P., Mootha V. K., Mukherjee S., Ebert B. L., Gillette M. A., et al (2005). Gene set enrichment analysis: a knowledge-based approach for interpreting genome-wide expression profiles. Proc Natl Acad Sci U S A, 102, 15545–15550. 10.1073/pnas.0506580102 16199517 PMC1239896

[32] Huang DW, Sherman BT, Lempicki RA. Bioinformatics enrichment tools: paths toward the comprehensive functional analysis of large gene lists. Nucleic Acids Res. 2009;37(1):1–13. 10.1093/nar/gkn923 19033363 PMC2615629

[33] Liberzon A., Subramanian A., Pinchback R., Thorvaldsdottir H., Tamayo P., & Mesirov J. P. (2011). Molecular signatures database (msigdb) 3.0. Bioinformatics, 27(12), 1739–1740. 10.1093/bioinformatics/btr260 21546393 PMC3106198

[34] Del Río-Moreno M, Alors-Pérez E, González-Rubio S, Ferrín G, Reyes O, Rodríguez-Perálvarez M, et al Dysregulation of the Splicing Machinery Is Associated to the Development of Nonalcoholic Fatty Liver Disease. J Clin Endocrinol Metab. 2019 8 1;104(8):3389–3402. 10.1210/jc.2019-00021 ; PMCID: PMC6590982. 30901032 PMC6590982

[35] Blijlevens M., van der Meulen-Muileman I.H., de Menezes R.X. et al High-throughput RNAi screening reveals cancer-selective lethal targets in the RNA spliceosome. Oncogene 38, 4142–4153 (2019). 10.1038/s41388-019-0711-z 30705407

[36] Turner N., Grose R. Fibroblast growth factor signaling: from development to cancer. Nat Rev Cancer 10, 116–129 (2010). 10.1038/nrc2780 20094046

[37] Helsten Teresa; Elkin Sheryl; Arthur Elisa; Tomson Brett; Carter Jennifer & Kurzrock, Razelle. (2015). The FGFR Landscape in Cancer: Analysis of 4,853 Tumors by Next-Generation Sequencing. Clinical cancer research: an official journal of the American Association for Cancer Research. 22 10.1158/1078-0432.CCR-14-3212 26373574

[38] Lee Joycelyn & Choo Su. (2018). The fibroblast growth factor receptor pathway in hepatocellular carcinoma. Hepatoma Research. 4 52 10.20517/2394–5079.2018.42

[39] Kim JM, Noh EM, Song HK, You YO, Jung SH, Kim JS, et al Silencing of casein kinase 2 inhibits PKC-induced cell invasion by targeting MMP-9 in MCF-7 cells. Mol Med Rep. 2018 6;17(6):8397–8402. 10.3892/mmr.2018.8885 Epub 2018 Apr 13. . 29658601

[40] Filhol O., Giacosa S., Wallez Y., et al Protein kinase CK2 in breast cancer: the CK2β regulatory subunit takes center stage in epithelial plasticity. Cell. Mol. Life Sci. 72, 3305–3322 (2015). 10.1007/s00018-015-1929-8 25990538 PMC11113558

[41] Romanuik TL, Wang G, Holt RA, Jones SJ, Marra MA, Sadar MD. Identification of novel androgen-responsive genes by sequencing of LongSAGE libraries. BMC Genomics. 2009 10 15;10:476 10.1186/1471-2164-10-476 ; PMCID: PMC2766392. 19832994 PMC2766392

[42] Kolluru V, Tyagi A, Chandrasekaran B, Ankem M, Damodaran C. Induction of endoplasmic reticulum stress might be responsible for defective autophagy in cadmium-induced prostate carcinogenesis. Toxicol Appl Pharmacol. 2019 6 15;373:62–68. 10.1016/j.taap.2019.04.012 Epub 2019 Apr 16. ; PMCID: PMC6572785. 31002860 PMC6572785

[43] Matsuda S; Katsumata R; Okuda T; Yamamoto T; Miyazaki K & Senga Takeshi, et al (2000). Molecular cloning and characterization of human MAWD, a novel protein containing WD-40 repeats frequently overexpressed in breast cancer. Cancer research. 60 13–7. 10646843

