# Supplementary figures and images for: Improved cancer biomarkers identification using network-constrained infinite latent feature selection
Source: PLoS One. 2021 Feb 11;16(2):e0246668. doi: 10.1371/journal.pone.0246668 (PMC7877636; doi:10.1371/journal.pone.0246668)

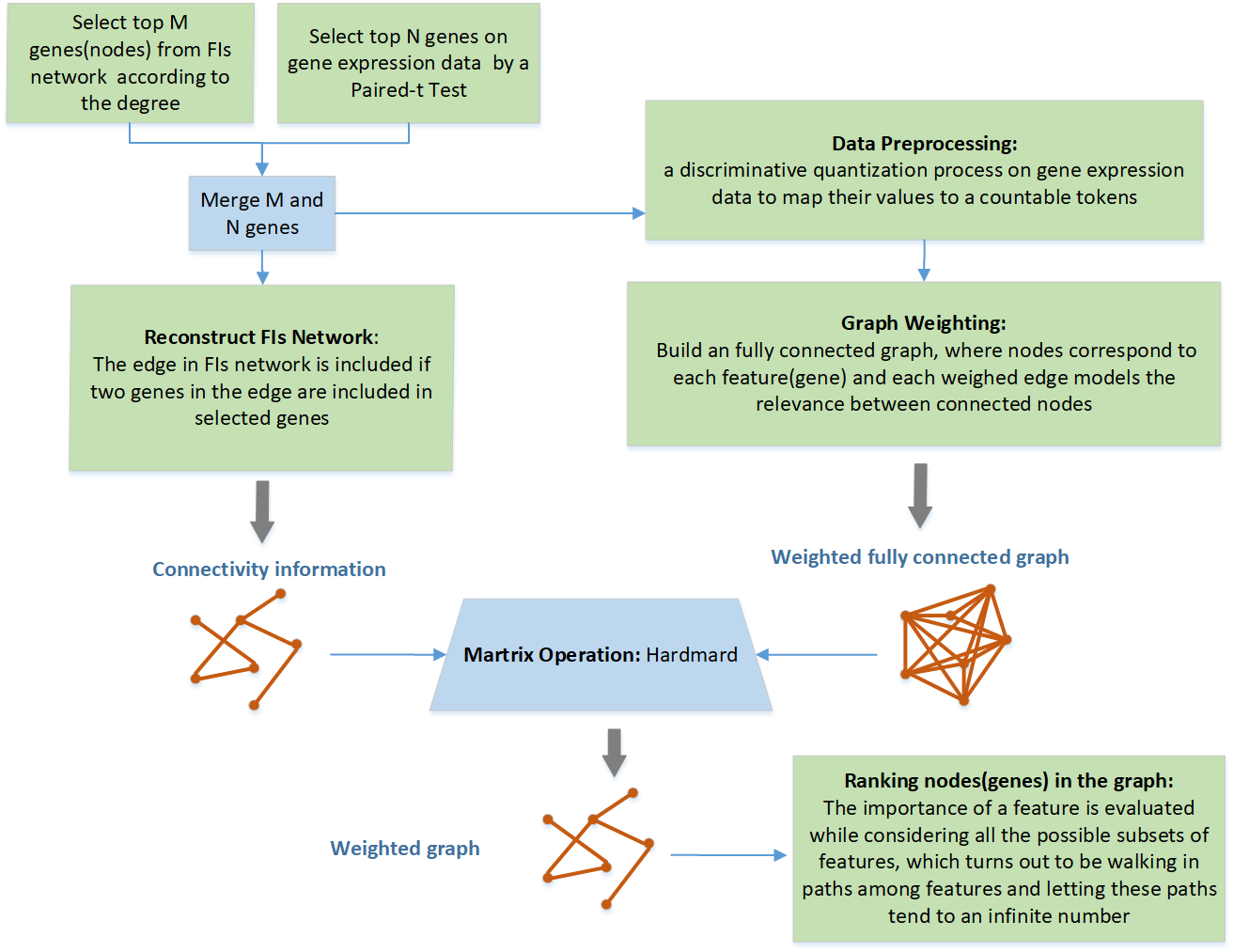

Supplement: S1 Fig — (TIF) [file pone.0246668.s001.tif]
